# A hybrid cuckoo search algorithm with Nelder Mead method for solving global optimization problems

**DOI:** 10.1186/s40064-016-2064-1

**Published:** 2016-04-18

**Authors:** Ahmed F. Ali, Mohamed A. Tawhid

**Affiliations:** Department of Computer Science, Faculty of Computers and Informatics, Suez Canal University, Ismailia, Egypt; Department of Mathematics and Statistics, Faculty of Science, Thompson Rivers University, 900 McGill Road, Kamloop, BC V2C 0C8 Canada; Department of Mathematics and Computer Science, Faculty of Science, Alexandria University, Moharam Bey Alexandria, 21511 Egypt

**Keywords:** Cuckoo search algorithm, Nelder–Mead method, Integer programming problems minimax problems

## Abstract

Cuckoo search algorithm is a promising metaheuristic population based method. It has been applied to solve many real life problems. In this paper, we propose a new cuckoo search algorithm by combining the cuckoo search algorithm with the Nelder–Mead method in order to solve the integer and minimax optimization problems. We call the proposed algorithm by hybrid cuckoo search and Nelder–Mead method (HCSNM). HCSNM starts the search by applying the standard cuckoo search for number of iterations then the best obtained solution is passing to the Nelder–Mead algorithm as an intensification process in order to accelerate the search and overcome the slow convergence of the standard cuckoo search algorithm. The proposed algorithm is balancing between the global exploration of the Cuckoo search algorithm and the deep exploitation of the Nelder–Mead method. We test HCSNM algorithm on seven integer programming problems and ten minimax problems and compare against eight algorithms for solving integer programming problems and seven algorithms for solving minimax problems. The experiments results show the efficiency of the proposed algorithm and its ability to solve integer and minimax optimization problems in reasonable time.

## Background

Cuckoo search (CS) is a population based meta-heuristic algorithm that was developed by Yang et al. (2007). CS (Garg 2015a, d) and other meta-heuristic algorithms such as ant colony optimization (ACO) (Dorigo 1992), artificial bee colony (Garg et al. 2013; Garg 2014; Karaboga and Basturk 2007), particle swarm optimization (PSO) (Garg and Sharma 2013; Kennedy and Eberhart 1995), bacterial foraging (Passino 2002), bat algorithm (Yang 2010a), bee colony optimization (BCO) (Teodorovic and DellOrco 2005), wolf search (Tang et al. 2012), cat swarm (Chu et al. 2006), firefly algorithm (Yang 2010b), fish swarm/school (Li et al. 2002), genetic algorithm (GA) (Garg 2015a), etc., have been applied to solve global optimization problems. These algorithms have been widely used to solve unconstrained and constrained problems and their applications. However, few works have been applied to solve minimax and integer programming problems via these algorithms.

A wide variety of real life problems in logistics, economics, social science, politics, game theory, and engineering can be formulated as integer optimization and minimax problems. The combinatorial problems, like the knapsack-capital budgeting problem, warehouse location problem, traveling salesman problem, decreasing costs and machinery selection problem, network and graph problems, such as maximum flow problems, set covering problems, matching problems, weighted matching problems, spanning trees problems, very large scale integration (LSI) circuits design problems, robot path planning problems, and many scheduling problems can also be solved as integer optimization and minimax problems (see, e.g., Chen et al. 2010; Du and Pardalos 2013; Hoffman and Padberg 1993; Little et al. 1963; Mitra 1973; Nemhauser et al. 1989; Zuhe et al. 1990).

Branch and bound (BB) is one of the most famous exact integer programming algorithm. However, BB suffers from high complexity, since it explores a hundred of nodes in a big tree structure when it solves a large scale problems. Recently, there are some efforts to apply some of swarm intelligence algorithms to solve integer programming problems such as ant colony algorithm (Jovanovic and Tuba 2011, 2013), artificial bee colony algorithm (Bacanin and Tuba 2012; Tuba et al. 2012), particle swarm optimization algorithm (Petalas et al. 2007), cuckoo search algorithm (Tuba et al. 2011) and firefly algorithm (Brown et al. 2007).

The minimax problem, as well as all other problems containing max (or min) operators, is considered to be difficult because max function is not differentiable. So many unconstrained optimization algorithms with the use of derivatives can not be applied to solve the non-differentiable unconstrained optimization problem directly.

There are several different approaches that have been taken to solve minimax problem. Many researchers have derived algorithms for the solution to minimax problem by solving an equivalent differentiable program with many constraints (see, e.g., Liuzzi et al. 2006; Polak 2012; Polak et al. 2003; Yang 2010b and the references therein), which may not be efficient in computing.

Some swarm intelligence (SI) algorithms have been applied to solve minimax problems such as PSO (Petalas et al. 2007). The main drawback of applying swarm intelligence algorithms for solving minimax and integer programming problems is the slow convergence and the expensive computation time for these algorithms.

Recent studies illustrate that CS is potentially far more efficient than PSO, GAs, and other algorithms. For example, in Yang et al. (2007), the authors showed that CS algorithm could outperform is very promising the existing algorithms such as GA and PSO. Also, CS algorithm has shown good performance both on benchmark unconstrained functions and applications (Gandomi et al. 2013; Yang and Deb 2013). Also, the authors in Singh and Abhay Singh (2014) compared latest metaheuristic algorithms such as Krill Herd algorithm (Gandomi and Alavi 2012), firefly algorithm and CS algorithm and found that CS algorithm is superior for both unimodal and multimodal test function in terms of optimization fitness and time processing.

Moreover, the CS algorithm has a few number of parameters and easy to implement which is not found on other meta-heuristics algorithms such as GA and PSO. Due to these advantage of the CS algorithm, many researchers have applied it on their work for various applications such as Garg et al. (2014), Garg (2015b, c, d). The CS algorithm is combined with other methods such as Nelder–Mead method to solve various problems (Chang et al. 2015; Jovanovic et al. 2014).

The aim of this work is to propose a new hybrid cuckoo search algorithm with a Nelder–Mead method in order to overcome the slow convergence of the standard cuckoo search. The Nelder–Mead method accelerates the search of the proposed algorithm and increases the convergence of the proposed algorithm. The proposed algorithm is called hybrid cuckoo search with Nelder–Mead (HCSNM). In HCSNM algorithm, we combine the cuckoo search with a Nelder Mead method in order to accelerate the search and avoid running the algorithm with more iterations without any improvements.

The main difference between our proposed algorithm and the other hybrid Cuckoo search and Nelder–Mead algorithms is the way of applying the Nelder–Mead method. The authors in Chang et al. (2015), Jovanovic et al. (2014) have invoked the Nelder–Mead method in the cuckoo search algorithm instead of the levy Flight operator. The drawback of this idea is the computation time because the calling for NM method at each iteration in the Cuckoo search algorithm. However in our proposed algorithm we run the standard CS algorithm for some iterations then we pass the best found solution to the Nelder–Mead method to start from good Solution which help the NM method to get the global minimum of the functions in reasonable time.

Also, we test the HCSNM algorithm on seven integer programming and ten minimax benchmark problems. The experimental results show that the proposed HCSNM is a promising algorithm and can obtain the optimal or near optimal solution for most of the tested function in reasonable time.

The outline of the paper is as follows. “Definition of the problems and an overview of the applied algorithms” section presents the definitions of the integer programming and the minimax problems and gives an overview of the Nelder–Mead method. “Overview of cuckoo search algorithm” section summarizes the main concepts of cuckoo search algorithm (CS). “The proposed HCSNM algorithm” section describes the main structure of the proposed HCSNM algorithm. “Numerical experiments” section gives the experimental results and details of implementation in solving integer programming and minimax problems. Finally, we end with some conclusions and future work in “Conclusion and future work” section.

## Definition of the problems and an overview of the applied algorithms

In this section, we present the definitions of the integer programming and the minimax problems as follows.

### The integer programming problem definition

An integer programming problem is a mathematical optimization problem in which all of the variables are restricted to be integers. The unconstrained integer programming problem can be defined as follows.

1 $$min f(x),\quad x \in S \subseteq {\mathbb{Z}}^n,$$

where ${\mathbb{Z}}$ is the set of integer variables, *S* is a not necessarily bounded set.

### Minimax problem definition

The general form of the minimax problem as reported in Yang (2010b) is defined by:

2 $$\text {min}\;F(x)$$

where

3 $$F(x)= \text {max}\;f_i(x), \quad i=1,\ldots ,m$$

with $$f_i(x):S\subset {\mathbb{R}}^n\rightarrow {\mathbb{R}}$$, $$i=1,\ldots ,m$$.

The nonlinear programming problems of the form:

$$\begin{aligned}&\text {min}\;F(x),\nonumber \\&g_i(x)\ge 0,\quad \;\;i=2,\ldots ,m,\nonumber \end{aligned}$$

can be transformed to minimax problems as follows:

4 $$\text {min}\;\text {max}\;f_i(x), \quad i=1,\ldots ,m$$

where

5 $$\begin{aligned}&f_1(x)=F(x), \\&f_i(x)=F(x)-\alpha _i g_i(x),\\&\alpha _i>0,\quad i=2,\ldots ,m \end{aligned}$$

It has been proved that for sufficiently large $\alpha _i$, the optimum point of the minimax problem, coincides with the optimum point of the nonlinear programming problem (Bandler and Charalambous 1974).

### Nelder Mead method

The Nelder–Mead algorithm (NM) is one of the most popular derivative-free nonlinear optimization algorithms. Nelder and Mead (1965) proposed NM algorithm. It starts with $n+1$ vertices (points) $x_1,x_2,\ldots ,x_{n+1}$. The vertices are evaluated, ordered and re-labeled in order to assign the best point and the worst point. In minimization optimization problems, the $x_1$ is considered as the best vertex or point if it has the minimum value of the objective function, while the worst point $x_{n+1}$ with the maximum value of the objective function. At each iteration, new points are computed, along with their function values, to form a new simplex. Four scalar parameters must be specified to define a complete NM algorithm: coefficients of reflection $\rho$, expansion $\chi$, contraction $\tau$, and shrinkage $\phi$ where $\rho > 0$, $\chi > 1$, $0< \tau < 1$, and $0< \phi < 1$. The main steps of the NM algorithm are presented as shown below in Algorithm 1. The vertices are ordered according to their fitness functions. The reflection process starts by computing the reflected point $x_r = {\bar{x}}+ \rho ({\bar{x}}- x_{(n+1)})$, where ${\bar{x}}$ is the average of all points except the worst. If the reflected point $x_r$ is lower than the *n*th point $f(x_{n})$ and greater than the best point $f(x_1)$, then the reflected point is accepted and the iteration is terminated. If the reflected point is better than the best point, then the algorithm starts the expansion process by calculating the expanded point $x_e = {\bar{x}} + \chi (x_r - {\bar{x}})$. If $x_e$ is better than the reflected point *n*th, the expanded point is accepted. Otherwise the reflected point is accepted and the iteration will be terminated. If the reflected point $x_r$ is greater than the *n*th point $x_n$ the algorithm starts a contraction process by applying an outside $x_{oc}$ or inside contraction $x_{ic}$ depending on the comparison between the values of the reflected point $x_r$ and the *n*th point $x_n$. If the contracted point $x_{oc}$ or $x_{ic}$ is greater than the reflected point $x_r$, the shrink process is starting. In the shrink process, the points are evaluated and the new vertices of simplex at the next iteration will be $x^{\prime }_2,\ldots ,x^{\prime }_{n+1}$, where $x^{\prime} = x_1 + \phi (x_i - x_1) , i = 2,\ldots , n + 1$.

## Overview of cuckoo search algorithm

In the following subsection, we summarize the main concepts and structure of the cuckoo search algorithm.

### Main concepts 

Cuckoo search algorithm is a population based metaheuristic algorithm inspired from the reproduction strategy of the cuckoo birds (Yang and Deb 2009). The cuckoo birds lay their eggs in a communal nests and they may remove other eggs to increase the probability of hatching their own eggs (Payne and Karen Klitz 2005). This method of laying the eggs in other nests is called obligate brood parasitism. Some host birds can discover the eggs are not their own and throw these eggs away or abandon their nest and build a new nest in a new place. Some kind of cuckoo birds can mimic the color and the pattern of the eggs of a few host bird in order to reduce the probability of discovering the intruding eggs. Since the cuckoo eggs are hatching earlier than the host bird eggs, the cuckoos laid their eggs in a nest where the host bird just laid its own eggs. Once the eggs are hatching, the cuckoo chick’s starts to propel the host eggs out the of the nest in order to increase its share of food provided by its host bird.

### L$\acute{e}$vy flights

Recent studies show that the behavior of many animals when searching for foods have the typical characteristics of L$\acute{e}$vy Flights, see, e.g., Brown et al. (2007), Pavlyukevich (2007) and Reynolds and Frye (2007). L$\acute{e}$vy flight (Brown et al. 2007) is a random walk in which the step-lengths are distributed according to a heavy-tailed probability distribution. After a large number of steps, the distance from the origin of the random walk tends to a stable distribution.

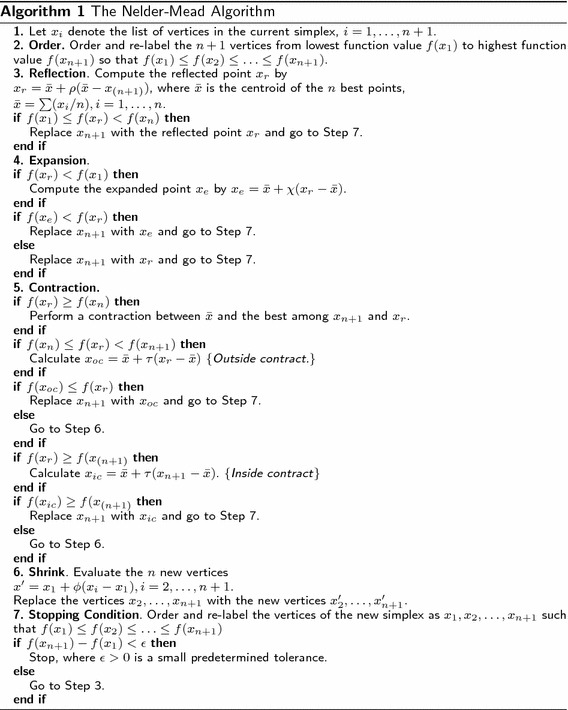



### Cuckoo search characteristic

The cuckoo search algorithm is based on the following three rules:

- At a time, cuckoo randomly chooses a nest to lay an egg.
- The best nests with high quality of eggs (solutions) will carry over to the next generations.
- The number of available host nests is fixed. The probability of discovering an intruding egg by the host bird is $p_a \in [0,1]$. If the host bird discovers the intruding egg, it throws the intruding egg away the nest or abandons the nest and starts to build a new nest elsewhere.

### Cuckoo search algorithm

We present in details the main steps of the Cuckoo search algorithm as shown in Algorithm 2.

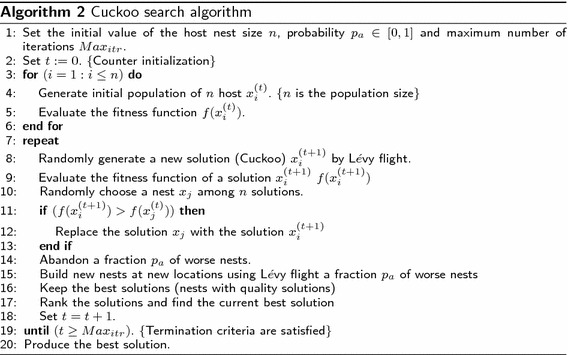

- *Step 1* The standard cuckoo search algorithm starts with the initial values of population size *n*, probability $p_a \in [0,1]$, maximum number of iterations $Max_{itr}$ and the initial iteration counter *t* (*Lines 1–2*).
- *Step 2* The initial population *n* is randomly generated and each solution $x_i$ in the population is evaluated by calculating its fitness function $f(x_i)$ (*Lines 3–6*).
- *Step 3* The following steps are repeated until the termination criterion is satisfied.
  - *Step 3.1* A new solution is randomly generated using a L${\acute{e}}$vy flight as follows.
    6 $$x_i^{t+1}=x_i^{t}+\alpha \oplus L\acute{e}vy(\lambda ),$$
    where $\oplus$ denotes entry-wise multiplication, $\alpha$ is the step size, and L${\acute{e}}$vy $(\lambda )$ is the L${\acute{e}}$vy distribution (*Lines 8–9*).
  - *Step 3.2* If its objective function is better than the objective function of the selected random solution, then the new solution is replaced with a random selected solution (*Lines 10–13*).
  - *Step 3.3* A fraction $(1-p_a)$ of the solutions is randomly selected, abandoned and replaced by new solutions generated via using local random walks as follows.
    7 $$x_i^{t+1}=x_i^{t}+\gamma \left( x_j^t-x_k^t\right) ,$$
    where $x_j^t$ and $x_k^t$ are two different solutions randomly selected and $\gamma$ is a random number (*Lines 14–15*).
  - *Step 3.4* The solutions are ranked according to their objective values, then the best solution is assigned. The iteration counter increases (*Lines 16–18*).
  - *Step 4* The operation is repeated until the termination criteria are satisfied (*Line 19*).
- *Step 6* Produce the best found solution so far (*Line 20*).

## The proposed HCSNM algorithm

The steps of the proposed HCSNM algorithm are the same steps of the standard CS algorithm till line 19 in Algorithm 2 then we apply the NM method in Algorithm 1 as an intensification process in order to refine the best obtained solution from the previous stage in the standard CS algorithm.

## Numerical experiments

In order to investigate the efficiency of the HCSNM, we present the general performance of it with different benchmark functions and compare the results of the proposed algorithm against variant of particle swarm optimization algorithms. We program HCSNM via MATLAB and take the results of the comparative algorithms from their original papers. In the following subsections, we report the parameter setting of the proposed algorithm with more details and the properties of the applied test functions. Also we present the performance analysis of the proposed algorithm with the comparative results between it and the other algorithms.

### Parameter setting

In Table 1, we summarize the parameters of the HCSNM algorithm with their assigned values.

Parameter values are selected either based on the common settings in the literature or determined through our preliminary numerical experiments.

**Fig. 1:**
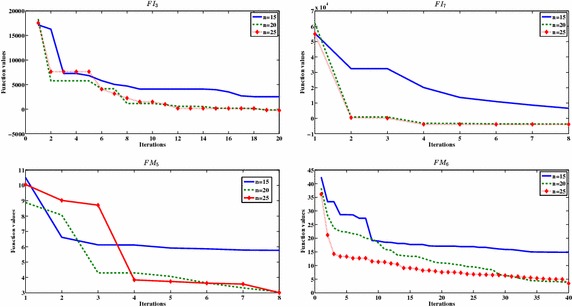
The effects of the number of population size

- *Population size n* The experimental tests show that the best population size is $n=20$, we applied the proposed algorithm with different population size in order to test the efficiency of the selected population size number. Figure 1 shows that the best population size is $n = 20,$ while increasing this number to $n = 25$ will increase the function evaluation without a big improvement in the function values.
- *A fraction of worse nests*$p_a$ In order to increase the diversification ability of the proposed algorithm, the worst solutions are discarded and the new solutions are randomly generated to replace the worst solutions. The number of the discarded solutions depends on the value of a fraction of worse nests $p_a$. The common $p_a$ value is 0.25.
- *Maximum number of iterations*$Max_{itr}$ The main termination criterion in standard cuckoo search algorithm is the number of iterations. In the proposed algorithm, we run the standard CS algorithm 3*d* iterations, then the best found solution is passed to the NM method. The effect of the maximum number of iteration is shown in Table 2. Table 2 shows that function values of six random selected functions (three integer functions and three minmax function). The results in Table 2 shows that there is no big different in the function value after applying 3*d* and 4*d* iterations which indicates that the number of iteration 3*d* is the best selection in term of function evaluation
- *Number of best solution for NM method*$N_{elite}$ In the final stage of the algorithm, the best obtained solution from the cuckoo search is refined by the NM method. The number of the refined solutions $N_{elite}$ is set to 1.

**Table 1:** Parameter setting

Parameters	Definitions	Values
*n*	Population size	20
$p_a$	A fraction of worse nests	0.25
$Max_{itr}$	Maximum number of iterations	3*d*
$N_{elite}$	No. of best solution for final intensification	1

**Table 2:** The effect of maximum number of iteration before applying Nelder–Mead method

Function	*d*	2*d*	3*d*	4*d*
$FI _1$	117.60	18.26	2.46	2.04
$FI _2$	2379.15	350.54	179.85	175.14
$FI _7$	870.11	1.014	0.0095	0.0042
$FM _3$	454.79	−39.14	−41.92	−41.93
$FM _6$	15.73	6.15	1.19	1.15
$FM _{10}$	459.25	1.05	0.114	0.114

### Integer programming optimization test problems

We test do the efficiency of the HCSNM algorithm by applying the algorithm on seven benchmark integer programming problems ($FI_1-FI_7$) as shown in Table 3. In Table 4, we list the properties of the benchmark functions (function number, dimension of the problem, problem bound and the global optimal of each problem). Now we define the test functions as follows. The solutions are rounded to the nearest integer for function evaluation purposes and they are consider as real numbers for all other operations.

**Table 3:** Integer programming optimization testproblems

Test problem	Problem definition
Problem 1 (Rudolph 1994)	$FI _{1}(x)=\Vert x\Vert _1=|x_1|+\cdots +|x_n|$
Problem 2 (Rudolph 1994)	$$FI _{2}(x)=x^Tx= \left[ \begin{array}{ccc} x_1&\ldots&x_n\end{array}\right] \left[ \begin{array}{c} x_1\\ \vdots \\ x_n\end{array}\right]$$
Problem 3 (GlankwahmdeeL et al. 1979)	$$FI _{3}(x)= \left[ \begin{array}{ccccc} 15&\quad 27&\quad 36&\quad 18&\quad 12 \end{array}\right] x +x^T \left[ \begin{array}{ccccc} 35&{}\quad -20&{}\quad -10&{}\quad 32&{}\quad -10\\ -20&{}\quad 40&{}\quad -6&{}\quad -31&{}\quad 32\\ -10&{}\quad -6&{}\quad 11&{}\quad -6&{}\quad -10\\ 32&{}\quad -31&{}\quad -6&{}\quad 38&{}\quad -20\\ -10&{}\quad 32&{}\quad -10&{}\quad -20&{}\quad 31\\ \end{array}\right] x$$
Problem 4 (GlankwahmdeeL et al. 1979)	$FI _{4}(x)=(9x_1^2+2x_2^2-11)^2+(3x_1+4x_2^2-7)^2$
Problem 5 (GlankwahmdeeL et al. 1979)	$FI _{5}(x)=(x_1+10x_2)^2+5(x_3-x_4)^2+(x_2-2x_3)^4+10(x_1-x_4)^4$
Problem 6 (Rao 1994)	$FI _{6}(x)=2x_1^2+3x_2^2+4x_1x_2-6x_1-3x_2$
Problem 7 (GlankwahmdeeL et al. 1979)	$FI _{7}(x)=-3803.84-138.08x_1-232.92x_2+123.08x_1^2+203.64x_2^2 + 182.25x_1x_2$

**Table 4:** The properties of the Integer programming test functions

Function	Dimension (d)	Bound	Optimal
$FI _1$	5	[−100 100]	0
$FI _2$	5	[−100 100]	0
$FI _3$	5	[−100 100]	−737
$FI _4$	2	[−100 100]	0
$FI _5$	4	[−100 100]	0
$FI _6$	2	[−100 100]	−6
$FI _7$	2	[−100 100]	−3833.12

### The efficiency of the proposed HCSNM algorithm with integer programming problems

In this subsection, we verify the importance of invoking the NM method in the final stage as a final intensification process. In Table 5, the results show the mean evaluation function values of the standard cuckoo search, the NM method and the proposed HCSNM algorithm, respectively. We apply the same termination criterion for all algorithms, which terminates the search when all algorithms reach to the optimal solution within an error of $10^{-4}$ before the 20,000 function evaluation value. We report the average function evaluation over 50 runs and give the best results in italicised text. The initial solution in the NM method is randomly generated. In Table 5, the results show that invoking the NM method in the final stage enhances the general performance of the proposed algorithm and can accelerate the search to reach to the optimal solution or near optimal solution.

**Table 5:** The efficiency of invoking the Nelder–Mead method in the final stage of SSSO algorithm for $FI_1 - FI_{7}$ integer programming problems

Function	Standard CS	NM method	HCSNM
$FI _1$	11,880.15	1988.35	*638*.*3*
$FI _2$	7176.23	678.15	*232*.*64*
$FI _3$	6400.25	*819*.*45*	1668.1
$FI _4$	4920.35	266.14	*174*.*04*
$FI _5$	7540.38	872.46	*884*.*48*
$FI _6$	4875.35	254.15	*155*.*89*
$FI _7$	3660.45	245.47	*210*.*3*

### The general performance of the HCSNM algorithm with integer programming problems

**Fig. 2:**
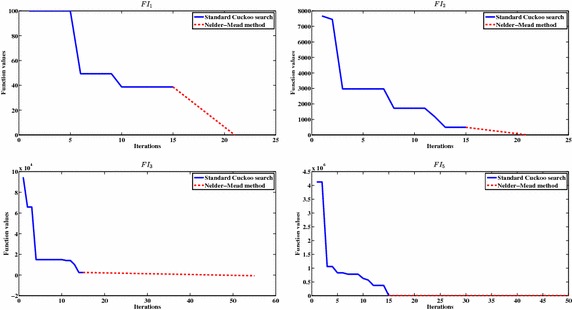
The general performance of the proposed HCSNM algorithm with integer problems

We apply the second experimental test to investigate the general performance of the proposed algorithm on the integer programming problems by plotting the values of function values versus the number of iterations as shown in Fig. 2 for four functions $FI_1, FI_2, FI_3$ and $FI_5$ (randomly picked). The solid line represents the standard cuckoo search algorithm, while the dotted line represents the performance of the NM method after applying he NM on the best obtained solution from the standard cuckoo search. We can conclude from Fig. 2 that invoking the NM method as an intensification process in the final stage of the proposed algorithm can accelerate the search and obtain the optimal or near optimal solution in reasonable time.

### HCSNM and other algorithms

We compare HCSNM with four benchmark algorithms (particle swarm optimization with its variants) in order to verify of the efficiency of the proposed algorithm. Before we give the comparison results of all algorithms, let us describe the comparative four algorithms (Petalas et al. 2007).

- *RWMPSOg* RWMPSOg is random walk memetic particle swarm optimization (with global variant), which combines the particle swarm optimization with random walk (as direction exploitation).
- *RWMPSOl* RWMPSOl is random walk memetic particle swarm optimization (with local variant), which combines the particle swarm optimization with random walk (as direction exploitation).
- *PSOg* PSOg is standard particle swarm optimization with global variant without local search method.
- *PSOl* PSOl is standard particle swarm optimization with local variant without local search method.

#### Comparison between RWMPSOg, RWMPSOl, PSOg, PSOl and HCSNM for integer programming problems

In this subsection, we give the comparison results between our HCSNM algorithm and the other algorithms in order to verify of the efficiency of our proposed algorithm. We test the five comparative algorithms on seven benchmark functions and report the results. We take the results of the comparative algorithms from their original paper (Petalas et al. 2007). In Table 6, we report the minimum (min), maximum (max), average (mean), standard deviation (SD) and success rate (%Suc) of the evaluation function values over 50 runs. The run is considered successful if the algorithm reaches to the global minimum of the solution within an error of $10^{-4}$ before the 20,000 function evaluation value. We report the best results between the comparative algorithms in italicised text. The results in Table 6 shows that the proposed HCSNM algorithm succeeds in six of seven function, where function $FI_6$ is little bit better than the proposed algorithm, however the rate of success of the proposed algorithm is 100 % for all functions.

**Table 6:** Experimental results (min, max, mean, standard deviation and rate of success) of function evaluation for $FI_1 - FI_{7}$ test problems

Function	Algorithm	Min	Max	Mean	SD	Suc
$FI _1$	RWMPSOg	17,160	74,699	27,176.3	8657	50
	RWMPSOl	24,870	35,265	30,923.9	2405	50
	PSOg	14,000	261,100	29,435.3	42,039	34
	PSOl	27,400	35,800	31,252	1818	50
	HCSNM	626	650	*638.3*	4.34	50
$FI _2$	RWMPSOg	252	912	578.5	136.5	50
	RWMPSOl	369	1931	773.9	285.5	50
	PSOg	400	1000	606.4	119	50
	PSOl	450	1470	830.2	206	50
	HCSNM	208	238	*232.64*	4.28	50
$FI _3$	RWMPSOg	361	41,593	6490.6	6913	50
	RWMPSOl	5003	15,833	9292.6	2444	50
	PSOg	2150	187,000	12,681	35,067	50
	PSOl	4650	22,650	11,320	3803	50
	HCSNM	1614	1701	*1668.1*	43.2	50
$FI _4$	RWMPSOg	76	468	215	97.9	50
	RWMPSOl	73	620	218.7	115.3	50
	PSOg	100	620	369.6	113.2	50
	PSOl	120	920	390	134.6	50
	HCSNM	163	191	*174.04*	6.21	50
$FI _5$	RWMPSOg	687	2439	1521.8	360.7	50
	RWMPSOl	675	3863	2102.9	689.5	50
	PSOg	680	3440	1499	513.1	43
	PSOl	800	3880	2472.4	637.5	50
	HCSNM	769	1045	*884.48*	56.24	50
$FI _6$	RWMPSOg	40	238	*110.9*	48.6	50
	RWMPSOl	40	235	112	48.7	50
	PSOg	80	350	204.8	62	50
	PSOl	70	520	256	107.5	50
	HCSNM	139	175	155.89	5.16	50
$FI _7$	RWMPSOg	72	620	242.7	132.2	50
	RWMPSOl	70	573	248.9	134.4	50
	PSOg	100	660	421.2	130.4	50
	PSOl	100	820	466	165	50
	HCSNM	119	243	*210.3*	6.39	50

#### HCSNM and other meta-heuristics and swarm intelligence algorithms for integer programming problems

We test the HCSNM algorithm with different meta-heuristics algorithms such as GA (Holland 1975), PSO (Kennedy and Eberhart 1995), firefly (FF) algorithm (Yang 2010b) and grey wolf optimizer (GWO) (Mirjalili et al. 2014). In order to make a fair comparison we set the population size = 20 for all algorithms and the termination criteria for all algorithm are the same which are the algorithm reaches to the global minimum of the solution within an error of $10^{-4}$ before the 20,000 function evaluation value. We applied the standard parameter setting for all compared meta-heuristics algorithms. In Table 7, we report the average (Avg) and SD of all algorithms over 50 runs.

**Table 7:** HCSNM and other meta-heuristics algorithms for $FI_1 - FI_{7}$ integer programming problems

Function	GA	PSO	FF	GWO	HCSNM
$$FI _1$$					
Avg	1640.23	20,000	1617.13	860.45	*613.48*
SD	425.18	0.00	114.77	43.66	21.18
$$FI _2$$					
Avg	1140.15	17,540.17	834.15	880.25	*799.23*
SD	345.25	1054.56	146.85	61.58	41.48
$$FI _3$$					
Avg	4120.25	20,000	1225.17	4940.56	*764.15*
SD	650.21	0.00	128.39	246.89	37.96
$$FI _4$$					
Avg	1020.35	16,240.36	476.16	2840.45	*205.48*
SD	452.56	1484.96	31.29	152.35	39.61
$$FI _5$$					
Avg	1140.75	13,120.45	1315.53	1620.65	*792.56*
SD	245.78	1711.83	113.01	111.66	53.32
$$FI _6$$					
Avg	1040.45	1340.14	345.71	3660.25	*294.53*
SD	115.48	265.21	35.52	431.25	33.90
$$FI _7$$					
Avg	1060.75	1220.46	675.48	1120.15	*222.35*
SD	154.89	177.19	36.36	167.54	33.55

Italic values indicate the best values

### HCSNM and the branch and bound method

We apply further investigation to verify of the powerful of the proposed algorithm with the integer programming problems, by comparing the HCSNM algorithm against the branch and bound (BB) method (Borchers and Mitchell 1991, 1994; Lawler and Wood 1966; Manquinho et al. 1997).

#### Comparison between the BB method and HCSNM for integer programming problems

In Table 8, we show the comparison results between the BB method and the proposed HCSNM. We take the results of the BB method from its original paper (Laskari et al. 2002). In Laskari et al. (2002), the BB algorithm transforms the initial integer problem programming problem to a continuous one. For the bounding, the BB uses the sequential quadratic programming method to solve the generated sub problems. While for branching, BB uses depth first traversal with backtracking. We report the average (Mean), SD and rate of success (Suc) over 30 runs. We report the best mean evaluation values between the two algorithms in italicised text. The results in Table 8 shows that the proposed algorithm results are better than the results of the BB method in six of seven tested functions, while the rate of success is 100 % for all function in the proposed algorithm. The overall results in Table 8 shows that the proposed algorithm is faster and more efficient than the BB method for most cases.

**Table 8:** Experimental results (mean, standard deviation and rate of success) of function evaluation between BB and HCSNM for $FI_1 - FI_{7}$ test problems

Function	Algorithm	Mean	SD	Suc
$FI _1$	BB	1167.83	659.8	30
	HCSNM	*638*.*26*	4.41	30
$FI _2$	BB	*139*.*7*	102.6	30
	HCSNM	230.86	4.68	30
$FI _3$	BB	4185.5	32.8	30
	HCSNM	*1670*.*5*	39.90	30
$FI _4$	BB	316.9	125.4	30
	HCSNM	*173*.*73*	5.57	30
$FI _5$	BB	2754	1030.1	30
	HCSNM	*898*.*3*	66.54	30
$FI _6$	BB	211	15	30
	HCSNM	*150*.*63*	3.10	30
$FI _7$	BB	358.6	14.7	30
	HCSNM	*211*.*1*	5.20	30

### Minimax optimization test problems

We consider another type of optimization test problems in order to investigate the efficiency of the proposed algorithm, these functions are ten benchmark minimax functions as shown in Table 9. We report their properties in Table 10.

**Table 9:** Minimax optimization test problems

Test problem	Problem defination
Problem 1 (Yang 2010b)	$FM _{1}(x)=\text {max}\;{f_i(x)},\; i=1,2,3,$
	$$f_1(x)=x_1^2+x_2^4,$$
	$$f_2(x)=(2-x1)^2+(2-x_2)^2,$$
	$$f_3(x)=2exp(-x_1+x_2)$$
Problem 2 (Yang 2010b)	$FM _{2}(x)=\text {max}\;{f_i(x)}, \;i=1,2,3,$
	$$f_1(x)=x_1^4+x_2^2$$
	$$f_2(x)=(2-x1)^2+(2-x_2)^2,$$
	$$f_3(x)=2exp(-x_1+x_2)$$
Problem 3 (Yang 2010B)	$FM _{3}(x)=x_1^2+x_2^2+2x_3^2+x_4^2-5x_1-5x_2-21x_3+7x_4,$
	$$g_2(x)=-x_1^2-x_2^2-x_3^3-x_4^2-x_1+x_2-x_3+x_4+8,$$
	$$g_3(x)=-x_1^2-2x_2^2-x_3^2-2x_4+x_1+x_4+10,$$
	$$g_4(x)=-x_1^2-x_2^2-x_3^2-2x_1+x_2+x_4+5$$
Problem 4 (Yang 2010B)	$FM _{4}(x)=\text {max}\;{f_i(x)}\;i=1,\ldots ,5$
	$$f_{1}(x)=(x_1-10)^2+5(x_2-12)^2+x_3^4+3(x_4-11)^2 +10x_5^6+7x_6^2+x_7^4-4x_6x_7-10x_6-8x_7,$$
	$$f_2(x)=f_1(x)+10(2x_1^2+3x_2^4+x_3+4x_4^2+5x_5-127),$$
	$$f_3(x)=f_1(x)+10(7x_1+3x_2+10x_3^2+x_4-x_5-282),$$
	$$f_4(x)=f_1(x)+10(23x_1+x_2^2+6x_6^2-8x_7-196),$$
	$$f_5(x)=f_1(x)+10(4x_1^2+x_2^2-3x_1x_2+2x_3^2+5x_6-11x_7$$
Problem 5 (Schwefel 1995)	$FM _{5}(x)=\text {max}\;{f_i(x)},\;i=1,2,$
	$$f_1(x)=|x_1+2x_2-7|$$,
	$$f_2(x)=|2x_1+x_2-5|$$
Problem 6 (Schwefel 1995)	$FM _{6}(x)=\text {max}\;{f_i(x)},$
	$$f_i(x)=|x_i|,\;i= 1,\ldots ,10$$
Problem 7 (Lukšan and Vlcek 2000)	$FM _{7}(x)= \text {max}\;{f_i(x)},\;i=1,2,$
	$$f_1(x)=(x_1-\sqrt{(x_1^2+x_2^2)}cos\sqrt{x_1^2+x_2^2})^2+0.005(x_1^2+x_2^2)^2,$$
	$$f_2(x)=(x_2-\sqrt{(x_1^2+x_2^2)}sin\sqrt{x_1^2+x_2^2})^2+0.005(x_1^2+x_2^2)^2$$
Problem 8 (Lukšan and Vlcek 2000)	$FM _{8}(x)= \text {max}\;{f_i(x)},\;i=1,\ldots ,4,$
	$$f_1(x)=(x_1-(x_4+1)^4)^2+(x_2-(x_1-(x_4+1)^4)^4)^2 +2x_3^2+x_4^2-5(x_1-(x_4+1)^4)-5(x_2-(x1-(x_4+1)^4)^4)-21x_3+7x_4,$$
	$$f_2(x)=f_1(x)+10[(x_1-(x_4+1)^4)^2+(x_2-(x_1-(x_4+1)^4)^4)^2 +x_3^2+x_4^2+(x_1-(x_4+1)^4)-(x_2-(x_1-(x_4+1)^4)^4)+x_3-x_4-8],$$
	$$f_3(x)=f_1(x)+10[(x_1-(x_4+1)^4)^2+2(x_2-(x_1 -(x_4+1)^4)^4)^2+x_3^2+2x_4^2-(x_1-(x_4+1)^4)-x_4-10]$$
	$$f_4(x)=f_1(x)+10[(x_1-(x_4+1)^4)^2+(x_2-(x_1 -(x_4+1)^4)^4)^2+x_3^2+2(x_1-(x_4+1)^4)-(x_2-(x_1-(x_4+1)^4)^4)-x_4-5]$$
Problem 9 (Lukšan and Vlcek 2000)	$FM _{9}(x)= \text {max}\;{f_i(x)},\;i=1,\ldots ,5,$
	$$f_1(x)=(x_1-10)^2+5(x_2-12)^2+x_3^4+3(x_4-11)^2+10x_5^6+7x_6^2+x_7^4-4x_6x_7-10x_6-8x_7$$,
	$$f_2(x)=-2x_1^2-2x_3^4-x_3-4x_4^2-5x_5+127$$,
	$$f_3(x)=-7x_1-3x_2-10x_3^2-x_4+x_5+282$$,
	$$f_4(x)=-23x_1-x_2^2-6x_6^2+8x_7+196$$,
	$$f_5(x)=-4x_1^2-x_2^2+3x_1x_2-2x_3^2-5x_6+11x_7$$
Problem 10 (Lukšan and Vlcek 2000)	$FM _{10}(x)=\text {max}\;{|f_i(x)|}, \;i=1,\ldots ,21,$
	$$f_i(x)=x_1exp(x_3t_i)+x_2exp(x_4t_i)-\frac{1}{1+t_i},$$
	$$t_i=-0.5+\frac{i-1}{20}$$

**Table 10:** Minimax test functions properties

Function	Dimension (d)	Desired error goal
$FM _1$	2	1.95222245
$FM _2$	2	2
$FM _3$	4	−40.1
$FM _4$	7	247
$FM _5$	2	$10^{-4}$
$FM _6$	10	$10^{-4}$
$FM _7$	2	$10^{-4}$
$FM _8$	4	−40.1
$FM _9$	7	680
$FM _{10}$	4	0.1

### The efficiency of the proposed HCSNM algorithm with minimax problems

We apply another test to investigate the idea of invoking the NM method in the final stage as a final intensification process with the standard Cuckoo search algorithm. In Table 11, we show the mean evaluation function values of the standard cuckoo search algorithm, the NM method and the proposed HCSNM algorithm, respectively. We apply for all algorithms the same termination criterion, which terminates the search when both algorithms reach to the optimal solution within an error of $10^{-4}$ before the 20,000 function evaluation value. We report the average function evaluation over 100 runs and the best results in italicised text. Also we show in Table 11 that invoking the NM method in the final stage in the proposed algorithm enhance the general performance of it and can accelerate the search to reach to the optimal solution or near optimal solution faster than the standard Cuckoo search algorithm and the NM method.

**Table 11:** The efficiency of invoking the Nelder–Mead method in the final stage of HCSNM for $FM_1 - FM_{10}$ minimax problems

Function	Standard CS	NM method	HCSNM
$FM _1$	5375.25	1280.35	*705*.*62*
$FM _2$	6150.34	1286.47	*624*.*24*
$FM _3$	3745.14	1437.24	*906*.*28*
$FM _4$	11,455.17	19,147.15	*3162*.*92*
$FM _5$	5845.14	1373.15	*670*.*22*
$FM _6$	7895.14	18,245.48	*4442*.*76*
$FM _7$	11,915.24	1936.12	*1103*.*86*
$FM _8$	20,000	2852.15	*2629*.*36*
$FM _9$	14,754.14	19,556.14	*2724*.*78*
$FM _{10}$	6765.24	1815.26	*977*.*56*

### HCSNM and other algorithms

We compare HCSNM with three benchmark algorithms in order to verify of the efficiency of the proposed algorithm with minimax problems. Let us give a brief description about these comparative three algorithms.

- *HPS2* (Santo and Fernandes 2011) HPS2 is heuristic pattern search algorithm, which is applied for solving bound constrained minimax problems by combining the Hook and Jeeves (HJ) pattern and exploratory moves with a randomly generated approximate descent direction.
- *UPSOm* (Parsopoulos and Vrahatis 2005) UPSOm is unified particle swarm Optimization algorithm, which combines the global and local variants of the standard PSO and incorporates a stochastic parameter to imitate mutation in evolutionary algorithms.
- *RWMPSOg* (Petalas et al. 2007). RWMPSOg is random walk memetic particle swarm optimization (with global variant), which combines the particle swarm optimization with random walk (as direction exploitation).

#### Comparison between HPS2, UPSOm, RWMPSOg and HCSNM for minimax problems

In this subsection, we present the comparison results between our HCSNM algorithm and the other algorithms in order to verify of the efficiency of the proposed algorithm. We test the four comparative algorithms on ten benchmark functions, take the results of the comparative algorithms from their original paper (Santo and Fernandes 2011) and report the results. In Table 12, we report the average (Avg), sD and Success rate (%Suc) over 100 runs. The mark (–) for $FM_8$ in HPS2 algorithm and $FM_2$, $FM_8$ and $FM_9$ in RWMPSOg algorithm in Table 12 means that the results of these algorithms for these functions are not reported in their original paper. The run is considered successful if the algorithm reaches the global minimum of the solution within an error of $10^{-4}$ before the 20,000 function evaluation value. The results in Table 12, show that the proposed HCSNM algorithm succeeds in most runs and obtains the objective value of each function faster than the other algorithms, except for functions $FM_3$, $FM_6$, $FM_9$ and $FM_{10}$ the HPS2 results are better than the proposed algorithm. The dimensions for functions $FM_4$, *FM*6, *F*7, *FM*8 and *FM*9 is 7, 10, 2, 4 and 7 respectively, which increase the number of function evaluations beyond 20,000 when applied the NM method. The rate of success for these function can increase to 100 % if the function evaluation criterion bigger than 20,000.

**Table 12:** Evaluation function for the minimax problems $FM _{1}-FM _{10}$

Algorithm	Problem	Avg	SD	%Suc
HPS2	$FM _1$	1848.7	2619.4	99
	$FM _2$	635.8	114.3	94
	$FM _3$	*141.2*	*28.4*	37
	$FM _4$	8948.4	5365.4	7
	$FM _5$	772.0	60.8	100
	$FM _6$	*1809.1*	2750.3	94
	$FM _7$	4114.7	1150.2	100
	$FM _8$	–	–	–
	$FM _9$	*283.0*	*123.9*	64
	$FM _{10}$	*324.1*	173.1	100
UPSOm	$FM _1$	1993.8	853.7	100
	$FM _2$	1775.6	241.9	100
	$FM _3$	1670.4	530.6	100
	$FM _4$	12,801.5	5072.1	100
	$FM _5$	1701.6	184.9	100
	$FM _6$	18,294.5	2389.4	100
	$FM _7$	3435.5	1487.6	100
	$FM _8$	6618.50	2597.54	100
	$FM _9$	2128.5	597.4	100
	$FM _{10}$	3332.5	1775.4	100
RWMPSOg	$FM _1$	2415.3	1244.2	100
	$FM _2$	–	–	–
	$FM _3$	3991.3	2545.2	100
	$FM _4$	7021.3	1241.4	100
	$FM _5$	2947.8	257.0	100
	$FM _6$	18,520.1	776.9	100
	$FM _7$	1308.8	505.5	100
	$FM _8$	–	–	–
	$FM _9$	–	–	–
	$FM _{10}$	4404.0	3308.9	100
HCSNM	$FM _1$	*705.62*	14.721	100
	$FM _2$	*624.24*	20.83	100
	$FM _3$	906.28	98.24	100
	$FM _4$	*3162.92*	218.29	90
	$FM _5$	*670.22*	11.07	100
	$FM _6$	4442.76	87.159	95
	$FM _7$	*1103.86*	125.36	95
	$FM _8$	*2629.336*	84.80	75
	$FM _9$	2724.78	227.24	95
	$FM _{10}$	977.56	176.82	100

Italic values indicate the best values

#### HCSNM and other meta-heuristics and swarm intelligence algorithms for minmax problems

Also we compare the proposed HCSNM algorithm against the same meta-heuristics and swarm intelligence algorithms (SI) which described in “HCSNM and other meta-heuristics and swarm intelligence algorithms for integer programming problems” for integer problems. The average (Avg) and SD of all algorithms are reported over 100 runs as shown in Table 13.

**Table 13:** HCSNM and other meta-heuristics algorithms for $FM_1 - FM_{10}$ minmax problems

Function	GA	PSO	FF	GWO	HCSNM
$$FM _1$$					
Avg	1080.45	3535.46	1125.61	2940.2	*275.45*
SD	83.11	491.66	189.56	490.22	6.40
$$FM _2$$					
Avg	1120.15	20,000	785.17	3740.14	*260.53*
SD	65.14	0.00	31.94	712.19	21.60
$$FM _3$$					
Avg	1270.65	2920.15	695.54	1120.25	*262.15*
SD	95.26	269.48	50.03	417.04	15.68
$$FM _4$$					
Avg	2220.45	9155.35	1788.26	4940.35	*1704.28*
SD	488.45	649.12	118.09	313.60	36.63
$$FM _5$$					
Avg	1040.84	5680.17	582.52	3520.45	*265.54*
SD	55.89	937.44	86.77	946.36	12.01
$$FM _6$$					
Avg	20,000	20,000	13,692.13	2080.35	*1658.23*
SD	0.00	0.00	900.12	938.33	201.92
$$FM _7$$					
Avg	1120.25	5643.65	2685.25	1020.45	*177.23*
SD	65.89	4.3.22	610.07	219.90	12.72
$$FM _8$$					
Avg	1280.35	20,000	7659.45	1620.46	*1555.47*
SD	78.23	0.00	583.21	281.25	59.97
$$FM _9$$					
Avg	20,000	6220.25	8147.45	3760.54	*2732.15*
SD	0.00	727.44	1026.22	246.52	66.84
$$FM _{10}$$					
Avg	1080.65	6680.19	748.17	1630.4	*489.17*
SD	68.15	509.34	98.59	37.36	27.29

The results in Table 13 shows that the proposed HCSNM algorithm is outperform the other met-heuristics and swarm intelligence algorithm

#### HCSNM and SQP method

Another test for our proposed algorithm, we compare the HCSNM with another known method which is called sequential quadratic programming method (SQP) (Boggs and Tolle 1995; Fletcher 2013; Gill et al. 1981; Wilson et al. 1963).

We test the results of the two comparative algorithms on ten benchmark functions, take the results of the SQP algorithm from paper (Laskari et al. 2002) and report the results. In Table 14, we report the average (Avg), SD and success rate (%Suc) over 30 runs. The run is considered successful if the algorithm reaches the global minimum of the solution within an error of $10^{-4}$ before the 20,000 function evaluation value. The results in Table 14, show that the proposed HCSNM algorithm outperforms the SQP algorithm in seven of ten functions, while the results of SQP algorithm are better than our proposed algorithm for functions $FM_3$, $FM_5$ and $FM_6$. We can conclude from this comparison that the proposed HCSNM outperforms the SQP algorithm in most cases of tested minimax problems.

**Table 14:** Experimental results (mean, standard deviation and rate of success) of function evaluation between SQP and HCSNM for $FM_1 - FM_{10}$ test problems

Function	Algorithm	Mean	SD	Suc
$FM _1$	SQP	4044.5	8116.6	24
	HCSNM	*704*	11.84	30
$FM _2$	SQP	8035.7	9939.9	18
	HCSNM	*727.53*	22.07	30
$FM _3$	SQP	*135.5*	*21.1*	30
	HCSNM	913.43	92.11	30
$FM _4$	SQP	20,000	0.0	0.0
	HCSNM	*3112.46*	211.47	27
$FM _5$	SQP	*140.6*	38.5	30
	HCSNM	669.23	12.42	30
$FM _6$	SQP	*611.6*	200.6	30
	HCSNM	4451.9	89.87	26
$FM _7$	SQP	15,684.0	7302.0	10
	HCSNM	*1025.46*	8.55	24
$FM _8$	SQP	20,000	0.0	0.0
	HCSNM	*2629.93*	91.58	22
$FM _9$	SQP	20,000	0.0	0.0
	HCSNM	*2720.4*	222.77	24
$FM _{10}$	SQP	4886.5	8488.4	22
	HCSNM	*978.13*	183.49	30

Italic values indicate the best values

## Conclusion and future work

In this paper, a new hybrid cuckoo search algorithm with NM method is proposed in order to solve integer programming and minimax problems. The proposed algorithm is called hybrid cuckoo search and Nelder–Mead algorithm (HCSNM). The NM algorithm helps the proposed algorithm to overcome the slow convergence of the standard by refining the best obtained solution from the cuckoo search instead of keeping the algorithm running with more iterations without any improvements (or slow improvements) in the results. In order to verify the robustness and the effectiveness of the proposed algorithm, HCSNM has been applied on seven integer programming and ten minimax problems. The experimental results show that the proposed algorithm is a promising algorithm and has a powerful ability to solve integer programming and minimax problems faster than other algorithms in most cases.

In the future work, we will focus on the following directions:

- Apply the proposed algorithms on solving constrained optimization and engineering problems.
- Modify our proposed algorithm to solve other combinatorial problems, large scale integer programming and minimax problems.
